# Community-Acquired, Post-COVID-19, Methicillin-Resistant Staphylococcus aureus Pneumonia and Empyema

**DOI:** 10.7759/cureus.22121

**Published:** 2022-02-11

**Authors:** Cameron McCraw, Sierra Forbush, Kovid Trivedi

**Affiliations:** 1 Pulmonology and Critical Care, Western University of Health Sciences, Lebanon, USA; 2 Pulmonary and Critical Care, Western University of Health Sciences, Lebanon, USA; 3 Pulmonary/Critical Care Medicine, Salem Pulmonary Associates/Salem Health, Salem, USA

**Keywords:** post-covid-19 complication, coronavirus disease (covid-19), spontaneous bacterial empyema, secondary infection, mrsa pneumonia

## Abstract

The coronavirus disease 2019 (COVID-19) pandemic has presented unprecedented challenges to the healthcare system globally, with opportunistic and secondary infections being one of the biggest challenges. Most secondary infections occur as nosocomial infections due to exposure to multidrug-resistant organisms in healthcare facilities. Secondary bacterial pneumonia complicates the care of hospitalized COVID-19 pneumonia patients. We present the case of a 77-year-old male who was diagnosed with COVID-19 pneumonia about four weeks before the current presentation to the hospital and was treated symptomatically in the community setting. During workup, he was diagnosed with multifocal pneumonia and right-sided empyema caused by methicillin-resistant *Staphylococcus aureus* (MRSA). He underwent chest tube thoracostomy followed by intrapleural fibrinolysis along with targeted antibiotic therapy. He needed video-assisted thoracoscopy with decortication due to inadequate improvement with intrapleural fibrinolysis. This case is a rare presentation of a community-acquired MRSA lung infection that occurred after recovery from COVID-19 pneumonia. This case emphasizes the importance of monitoring for secondary infections, as well as highlights the extent of secondary infections in COVID-19.

## Introduction

Coronavirus disease 2019 (COVID-19) is caused by severe acute respiratory syndrome coronavirus 2 (SARS-CoV-2), a novel coronavirus that was initially announced to the World Health Organization (WHO) on December 31, 2019. It was first discovered in Wuhan, Hubei Province, China due to what was thought to be an unusual increase in suspected pneumonia cases. With rapid spread across the globe, WHO declared it a global pandemic on March 11, 2020 [1].

As of February 6, 2022, over 392 million cases and 5.7 million deaths have been reported globally [2]. The identification, diagnosis, and treatment of the disease have been hindered by the development of multiple variants, which have offered conglomerate challenges and overwhelmed healthcare systems worldwide.

Through increased surveillance, unique clinical presentations of post-COVID-19 opportunistic infections continue to be reported even after two years. However, the exact prevalence remains undetermined. An increase in the incidence of opportunistic fungal infections in COVID-19 patients has been noted, including the *Aspergillus* spp., *Candida* spp., and a group of Mucormycosis-causing molds. Parasitic opportunism in *Cryptococcus neoformans*, *Pneumocystis jiroveci*, *Strongyloides stercoralis*, and *Toxoplasma gondii* coinfection and post-infection have emerged as well. A meta-analysis showed that bacterial coinfections and secondary bacterial infections have been detected in 3.5% and 14.3%, respectively, of the COVID-19 patient population tested. They also found that the incidence of opportunistic infection and bacterial coinfection was more likely in those who are critically ill as well as had a longer length of hospital stay (95% confidence interval) [3]. A retrospective cohort study in New York corroborated the results to show that 158 of 4,221 patients admitted with COVID-19 had respiratory cultures performed. On day three of hospitalization, one (0.6%) patient of those who had respiratory cultures grew methicillin-resistant *Staphylococcus aureus* (MRSA), and by day 21 of hospitalization, 27 (5.7%) patients of those with respiratory cultures grew MRSA [4]. Although this might just show the prevalence, it defines the magnitude of transmission.

Post-COVID-19 opportunistic infections noted in emerging literature have been described to include various viral pathogens, most commonly including parainfluenzae and rhinoviruses, influenza, herpes simplex virus (HSV), and cytomegalovirus (CMV), with HSV and CMV being more common in immunosuppressed individuals [3]. Compared to previous HSV reactivation before COVID-19, that is, unrelated to COVID-19, the post-COVID-19 attacks were more severe in approximately 43% of the patients enrolled in the study. Interestingly, over one-third of the patients enrolled in the study reported having developed their very first symptomatic HSV attack during their active COVID-19 infection, suggesting a relationship between COVID-19 and HSV [5].

Most of the data come from hospitalized patients as secondary infections are more likely to be nosocomial. Here, we describe a case of post-COVID-19 MRSA pneumonia and empyema that developed in a patient who was never hospitalized for COVID-19 infection.

## Case presentation

A 77-year-old male was brought into the emergency department (ED) after a ground-level fall from his assisted living facility. He was transferring from his wheelchair to the toilet when his legs gave out and he fell. He hit his right flank on the toilet and did not lose consciousness. He had been experiencing generalized weakness for the past two to three months and had multiple mechanical falls in this duration. He complained of progressive shortness of breath and right-sided chest pain on deep breathing for the last week. There was no concurrent history of lightheadedness, dizziness, nausea, vomiting, vision changes, focal weakness, fever, chills, cough, or any other complaints. The patient was diagnosed with COVID-19 pneumonia about four weeks ago and, based on recommendations at that time, was quarantined at his assisted living facility with symptomatic management. The patient had not received COVID-19 vaccination or targeted monoclonal antibodies because they were not approved at the time. He had not been hospitalized in the last 90 days.

The patient had a history of coronary artery disease, type II diabetes mellitus, dementia, chronic systolic and diastolic heart failure, chronic constipation, osteoarthritis, gastroesophageal reflux disease, transient ischemic attack, hypertension, dyslipidemia, deep venous thrombosis, smoking, achalasia cardia, and alcohol use disorder. He was not an active alcoholic at the time of hospitalization. Surgical history was significant for coronary artery bypass grafting, appendectomy, and cholecystectomy.

Vitals in the ED were blood pressure 136/78 mmHg, pulse 105 beats per minute, respiratory rate 18 breaths per minute, temperature 99.3°F, and saturation 95% on room air. The patient’s saturation decreased to 90% with minimal activity. He looked chronically ill on appearance. Significant examination findings included unsteady gait, tender sacral decubitus ulcer, trace bilateral lower extremity edema, and bilateral basilar crackles. The rest of the physical examination was within normal limits.

**Figure 1:**
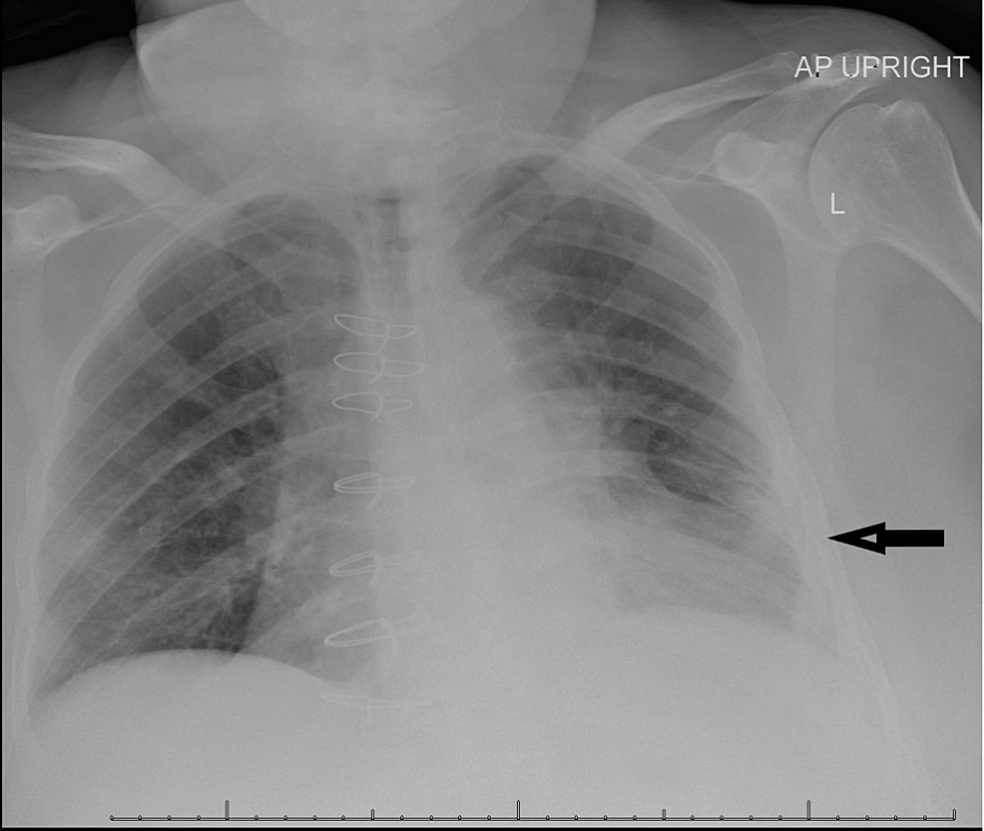
Chest radiograph showing left peripheral lower zone pleural thickening versus non-layering pleural effusion.

Laboratory findings showed troponin T <0.01 ng/mL, N-terminal pro-B-type natriuretic peptide 1,365 pg/ml, and a normal complete metabolic panel. Procalcitonin was elevated at 0.15 ng/mL. Complete blood count revealed normal hemoglobin and elevated total white blood cell count at 13,900/mm^3^ with a left shift (neutrophils 88%). Urinalysis was unremarkable. Chest radiograph showed left peripheral lower zone pleural thickening versus non-layering pleural effusion (Figure 1).

There was cardiomegaly. Additionally, there was no clinical or radiological evidence of a fracture. The last echocardiogram performed one year ago showed moderate concentric left ventricular hypertrophy. Left ventricular ejection fraction was 40-45% with indeterminate diastolic function. There was hypokinesis of the mid and distal septum, distal anterior wall, and apex. There was mild tricuspid regurgitation, as well as mild-to-moderate pulmonary regurgitation. The right ventricular systolic pressure was estimated at 29 mmHg.

**Figure 2:**
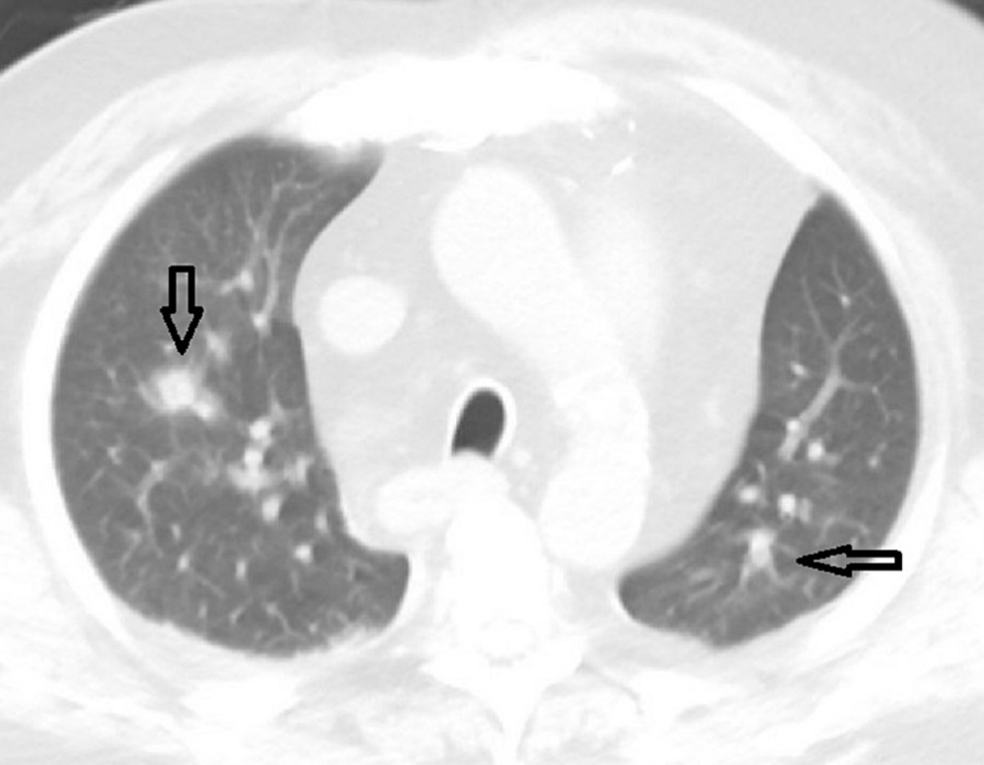
CT of the chest showing nodular multifocal consolidation bilaterally. CT: computerized tomography

The patient was admitted to the hospital with a working diagnosis of acute exacerbation of chronic heart failure. He was started on intravenous furosemide. Despite mild elevation in procalcitonin and leukocytosis with left shift, the clinical suspicion for infection was low, and antibiotics were deferred. The infiltrates were considered to be related to the recovery process from COVID-19 pneumonia. Despite adequate diuresis, the patient’s respiratory symptoms did not improve, and he underwent computerized tomography (CT) scan of the chest on the next day that showed nodular multifocal consolidation bilaterally (Figure 2) with lobar consolidation in the right lower lobe.

**Figure 3:**
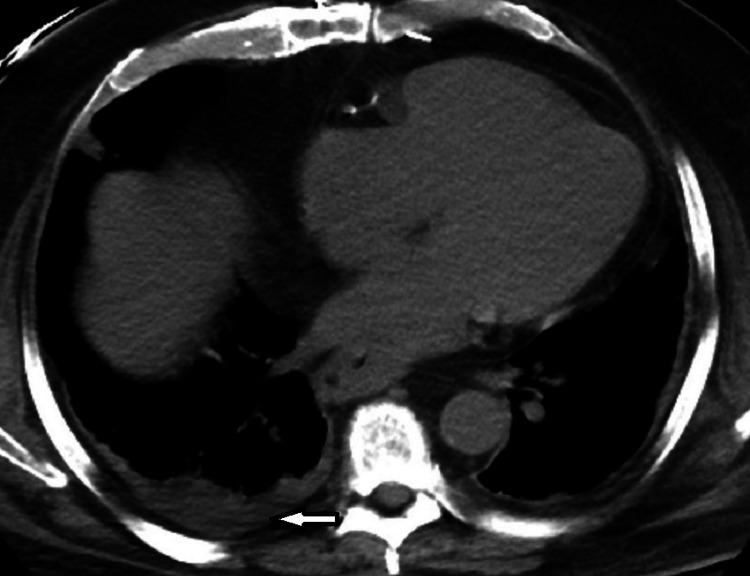
CT chest showing small right pleural effusion. CT: computerized tomography

There was a small right pleural effusion (Figure 3).

The patient had a thickened distal esophageal wall. He now admitted to dysphagia and a few vomiting episodes in the last few months. Based on this new information, aspiration pneumonia was thought to be the most plausible cause of his chest imaging findings, and he was started on broad-spectrum coverage with piperacillin-tazobactam. His clinical status did not show any improvement in the next two days, and a diagnostic thoracentesis was ordered to rule out empyema. Subsequently, 250 mL of orange-colored turbid pleural fluid was aspirated, and the analysis showed lactate dehydrogenase (LDH) 1,139 U/L, pH 7.56, and nucleated cell count 9,557/mm^3^ with 71% neutrophils. Gram stain of pleural fluid returned with gram-positive cocci the same day. Empiric vancomycin was added while awaiting cultures. With the diagnosis of empyema, a chest tube thoracostomy was performed. Pleural fluid cultures were positive for MRSA. Vancomycin was continued and piperacillin-tazobactam was stopped.

**Figure 4:**
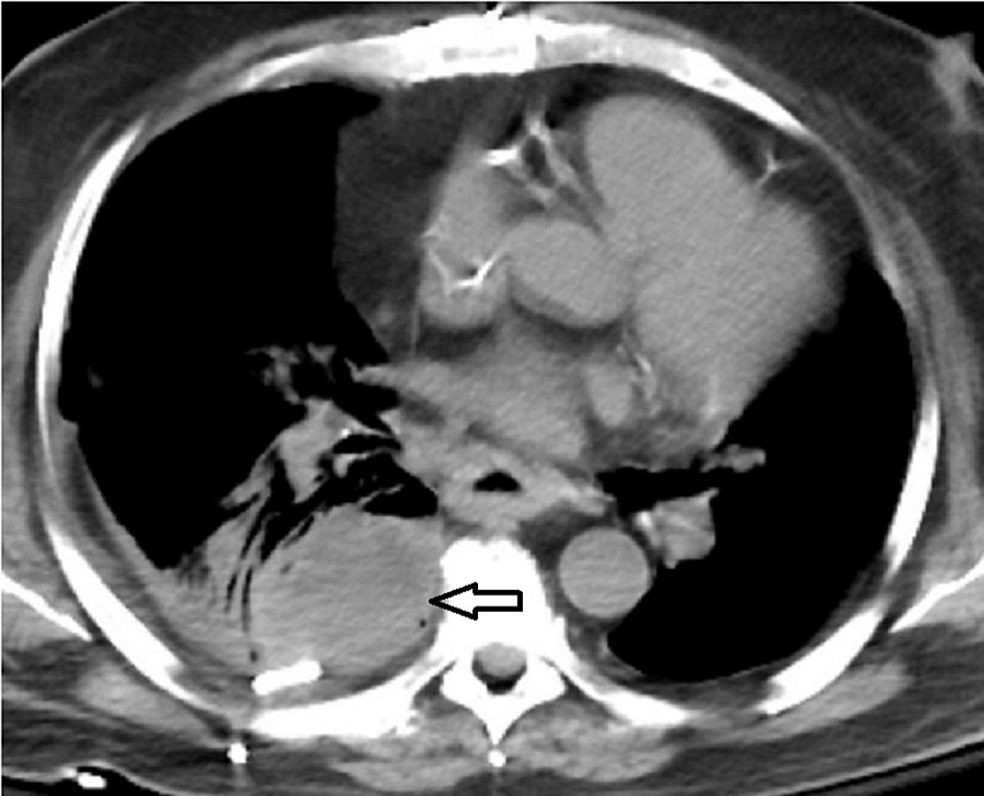
Follow-up CT showing persistent loculations in the right pleural space. CT: computerized tomography

Chest tube output was minimal, and repeat CT chest showed misplacement of the tube. It was removed and another chest tube was placed under CT guidance. Intrapleural fibrinolysis was started with alteplase and dornase instillation for a total of six sessions over three days. He had over 2 L of output through the chest tube over these three days. Follow-up CT still showed persistent loculations in the right pleural space (Figure 4).

Thoracic surgery was consulted, and the patient underwent video-assisted thoracoscopy with decortication. He had an unremarkable postoperative course and was treated with intravenous vancomycin for five weeks from the date of surgery.

## Discussion

COVID-19 infection can cause a reduction in immunity, which, in turn, can lead to secondary infections or coinfections. Researchers also suggest a potential association between COVID-19 infection and primary infection or reactivation, as seen with CMV and HSV, respectively. Pathogens that can become latent and reactivate during immunosuppression complicate diagnosis and treatment and are detrimental to patient health outcomes. While a majority of opportunistic infections and coinfections are mild in their clinical presentations, their pathology can be pronounced among the immunosuppressed. Patients undergoing a treatment regimen with immunosuppressive drugs such as glucocorticoids are at an increased risk of such opportunistic infection [3]. Our patient did not receive any glucocorticoids during his COVID-19 treatment.

Pre-pandemic total nosocomial infections incidence varied from 3.5% to 12%, and the WHO claimed an average incidence of 8.7% across 55 hospitals in 14 countries. A retrospective study in China examined over 250 COVID-19 patients which revealed coinfection with 24 respiratory pathogens. The highest incidences of bacterial coinfection included *Streptococcus pneumoniae*, *Klebsiella pneumoniae*, and *Hemophilus influenzae* [6]. Secondary infections are associated with a significant increase in morbidity and mortality, and researchers suggest approximately half of COVID-19 deaths can be attributed to such secondary infections. These patients stay in intensive care units longer and thus are subject to subsequent nosocomial infection. Such secondary infections increase their risk of sepsis, and it follows that they are then treated with antimicrobials, which may enhance nosocomial infections by multidrug-resistant organisms. This has also been demonstrated in urban India where secondary infections in COVID-19 patients with antibiotic-resistant bacteria have been the country’s biggest challenge at mitigating mortality [7].

Screening and treatment strategies targeted toward coinfections will improve patient outcomes. A meta-analysis of 20 studies, with a total of 205,702 patients, demonstrated that patients with tuberculosis and influenza have an especially increased risk of mortality if coinfected with COVID-19; however, the same report showed no significant increase in mortality found in other chronic illnesses, human immunodeficiency virus, or hepatitis (95% CI) [8]. It is also seen that those with concurrent bacterial infections patients sometimes develop a serious COVID-19 complication, called novel coronavirus-infected pneumonia. It is described as an atypical pneumonia with diffuse bilateral lung involvement which can progress to acute lung injury and, if severe, acute respiratory distress syndrome [9]. This pathology increases the chance of respiratory failure, shock, multiple organ failure, cytokine storm, coagulopathy, and death, particularly in patients who are immunosuppressed. Other risk factors to a worsening respiratory prognosis include cardiovascular disease, diabetes mellitus, hypertension, obesity, high inflammatory markers (particularly interleukin-6 and C-reactive protein), and chronic kidney disease [3].

Post-influenza pneumonia with positive MRSA cultures was shown to occur in nearly 6% of the patients enrolled in a recent review [4]. Our case shows a rare presentation of post-COVID-19 MRSA pneumonia with empyema as a peculiar characteristic. Because the patient was treated as an outpatient for COVID-19, the infection was non-nosocomial in nature and opportunistic. The emergence of novel observations calls for additional reports of unique COVID-19 clinical presentations, particularly if they are characterized by opportunistic or secondary infections that may serve as a treatment challenge. The impact of opportunistic and superadded infections is compounded even more by the recent development of COVID-19 variants. This calls for the enhancement of existing strategies to expedite the diagnosis process to identify COVID-19 and its variants earlier. This may also allow for earlier treatment which may serve as prophylaxis to its complications. Screening algorithms should be developed to identify who is at risk for these complications. Prospective studies on superinfection are warranted and should include data on nosocomial and non-nosocomial infections separately to gauge the burden clearly.

## Conclusions

This case emphasizes the importance of secondary infections in post-viral pneumonia situations. Although these infections are more common in nosocomial settings, community-acquired secondary infections are also increasing. A high index of suspicion for drug-resistant organisms is needed based on the community prevalence of these isolates. Post-Influenza MRSA pneumonia is a well-defined entity, but post-COVID-19 MRSA pneumonia has not been well described in the literature.
